# Effect of Elderly School Policy on Quality of Life among Thailand’s Senior Citizens: A Propensity Score Matching Approach

**DOI:** 10.12688/f1000research.151221.2

**Published:** 2025-03-31

**Authors:** Worapath Kratoo, Nuchanad Hounnaklang

**Affiliations:** 1 College of Public Health Sciences, Chulalongkorn University, Bangkok, Bangkok, 10330, Thailand

**Keywords:** Elderly school, Health policy, Quality of life, Propensity score matching

## Abstract

**Background:**

As Thailand’s population ages, promoting senior citizens’ quality of life (QoL) is crucial. In 2017, the Ministry of Social Development and Human Security launched the “elderly school” initiative to foster lifelong learning and enhance the QoL among senior citizens. However, comprehensive evaluations of its impact on QoL remain limited.

**Methods:**

This cross-sectional survey aimed to assess the policy’s effect on QoL in Phetchabun province, Thailand. Using quota and systematic sampling, 1,374 senior citizens aged 60-80 participated. Propensity score matching (PSM) with a 1:1 match was employed to estimate the average treatment effect (ATE) of attending the elderly school on QoL. Additionally, multiple linear regression was analyzed to assess the association between QoL and its associated factors.

**Results:**

PSM were matched successfully, the standardized difference was less than 10 percent, and the baseline after matching indicated balances with 687 elderly people in each group. The mean QoL score of the non-attending group was 44.40 (SD = 7.11), and that of the attending group was 57.50 (SD = 7.53). The ATE for elderly people attending school was 10.67 scores (95% CI: 9.67 – 11.67 scores) higher than those unattended. Being female, having monthly income higher than 20,000, having employment, having a caregiver, and attendance at elderly school were positively associated with QoL, and the standardized beta coefficients were 0.078, 0.059, 0.094, 0.066, and 0.550, respectively. Additionally, higher education was positively associated with higher QoL.

**Conclusion:**

The elderly school policy significantly enhanced the QoL of the attending senior citizens. Findings suggest continued collaboration among stakeholders to sustain and optimize this policy for improved seniors’ QoL, which has the potential to utilize lifelong learning to create an inclusive framework for healthy aging among senior citizens.

## Introduction

A global surge in the elderly population, defined as those aged 60 and above, presents a major challenge and opportunity for healthcare systems worldwide. From 2015 to 2050, the proportion of elders is projected to nearly double, from 12% to 22% ^1^, with 80% residing in middle-income countries. ^2^ This trend is particularly evident in Thailand, where the elderly population is expected to reach 17 million by 2040, exceeding one-quarter of the national total. ^3^^,^^4^ This necessitates immediate attention to preparing and evolving healthcare systems to adequately support the QoL of senior citizens.

Thailand, with its rapidly aging population, stands at the forefront of this global challenge. By acknowledging the urgency of the situation and proactively adapting its healthcare systems, Thailand can not only serve as a model for other countries but also ensure the health and QoL of its senior citizens, paving the way for a brighter future for all. ^5^^,^^6^

The concept of QoL encompasses an individual’s or population’s overall QoL, integrating both positive and negative experiences over a defined timeframe. ^7^ The World Health Organization (WHO) defines QoL as an individual’s subjective perception of their life position, influenced by cultural and value systems. ^8^^,^^9^ This multidimensional concept, as outlined by the WHO, encompasses four key domains: physical health, mental health, social relationships, and the environment. ^10^ QoL serves as a crucial indicator for achieving Sustainable Development Goals (SDGs), particularly for the elderly population, with relevance to SDG 3: Good Health and Well-being. ^11^

Despite achieving a moderate average QoL amongst senior citizens nationwide, Thailand faces disparities in urban-rural QoL, with literature suggesting consistently lower QoL for rural older adults ^12^^–^^14^ which is similar to Myanmar, and South India ^15^^,^^16^ This presents a significant challenge for Lomkao district, Phetchabun province, a rural area boasting a 24.8% elderly population and exhibiting moderate QoL scores for elder residents. ^17^^,^^18^ Research points beyond individual characteristics such as age, income, education, and chronic health conditions as sole determinants of QoL for older adults. Living arrangements and social interactions have also emerged as crucial factors deserving consideration in an effort to enhance elderly QoL. ^19^^–^^21^ Therefore, a comprehensive approach addressing both individual and environmental factors is essential to effectively improve QoL for Thailand’s rural elderly population.

In response to the crucial role of health promotion interventions in improving and maintaining QoL for older adults, numerous such programs have been implemented globally. ^22^^–^^24^ These interventions typically share three core objectives: enhancing functional capacity, promoting self-care behaviours, and preventing or delaying chronic illness onset. ^25^ Implementation often involves collaboration among social welfare organizations, healthcare professionals, and educational systems. ^26^^,^^27^ Thailand exemplifies this trend with the 2017 establishment of the elderly school initiative by the Department of Older Persons, Ministry of Social Development and Human Security. ^28^ This program, grounded in the concepts of awareness, empowerment, and lifelong learning, aims to equip older adults with the knowledge and skills necessary to achieve optimal QoL. ^29^^,^^30^

In 2018, Lomkao district was identified as one of 73 districts in Thailand by the District Health Board for the implementation of specific programs to address the challenges posed by an aging population. Recognizing the district’s demographic shift, the Board selected Lomkao as a priority area due to the prevalence of older adults residing within its boundaries. One of the key initiatives implemented in Lomkao to address this demographic trend is the “Elderly School” program. This program represents a targeted intervention aimed at improving the well-being and QoL for older adults in the district. ^31^^,^^32^ As of 2022, Thailand boasts a network of 2,370 elderly schools established through partnerships with local administrative organizations (LAOs), private entities, and community groups. ^33^ These schools offer a core curriculum encompassing four key modules: health, social, economic, and environmental education, in which the officers invite elderly people in communities to attend through voluntary participation. ^28^^,^^34^^,^^35^ Program adaptations cater to specific regional contexts, aligning with the curriculum framework developed by the Department of Older Persons (DOP), Ministry of Social Development and Human Security. Annual budgetary allocations for elderly school support have ranged from $283,700 to $425,687 USD over the past five years. Notably, the 2021 allocation fell below the minimum due to the COVID-19 pandemic necessitating online instruction in some areas where there is hardly any regular class. ^33^^,^^36^^–^^39^

Recognizing the program’s value, alternative funding avenues through LAOs, the Community Health Security Fund, and the Thai Health Promotion Foundation have been established to bolster implementation initiatives. ^40^ Beyond budgetary considerations, elderly schools demonstrably contribute to active aging, improved QoL, and ongoing learning among senior citizens. They provide valuable social interaction, alleviating loneliness and fostering a sense of community. Moreover, they empower older adults to productively utilize their free time, contributing to a sense of agency and QoL. ^41^^,^^42^

The establishment of the Elderly School initiative in Thailand in 2017 aimed to address the growing needs of the nation’s aging population. However, despite its implementation across various districts, a comprehensive and dedicated assessment of its impact on senior citizens’ QoL remains elusive. This lack of evaluation presents a significant gap in understanding the effectiveness of the program and its potential for improvement. Existing research has primarily focused on the program’s development and limitations of design, which could not control the influence of covariates. This might lead to a selection bias, leaving a critical gap in understanding its effectiveness. ^43^^–^^46^ To address this void, the present study employs PSM to evaluate the initiative’s causal effect on QoL within an aging society. Through PSM, we compare the QoL of two groups: senior citizens not attending and attending elderly schools. This approach facilitates the creation of comparable groups in real-world settings, mimicking an experimental study while mitigating selection bias and strengthening the robustness of regression analyses. In addition, investigating the association between various factors and QoL. Our findings aim to contribute to the existing body of literature, providing valuable guidance for the future implementation and refinement of lifelong learning initiatives for senior citizens in Thailand.

## Methods

### Research design

This cross-sectional study was conducted in Lomkao, Phetchabun province, Thailand between December 2022 to June 2023.

### Sample size

The study population consisted of 11,254 elderly individuals aged 60–80 years in Lomkao district, Phetchabun province, Thailand. ^17^ The sample size was calculated for the continuous data since this study analyzed multiple linear regression. A pilot test comparing the QoL between elderly individuals who attended and did not attend elderly school indicated a small effect size of 0.02, a power of 0.8, 13 predictors, and a significance level of 0.05. ^47^ There are 901 elderly people, with a minimum of 451 participants in each group. Inclusion criteria were 1) age between 60 and 80 years 2) residency in Lomkao, Phetchabun province, for at least one year and 3) literacy and proficiency in Thai communication (listening and speaking). The exclusion criteria focused on individuals with mental health illnesses that include anxiety disorders, mood disorders, and physical disabilities that would significantly impede their ability to participate in the study activities.

### Sampling technique

In this study, researchers utilized a combination of quota and systematic sampling techniques. The quota sampling was based on a 1:1 matching model, comparing elderly people who attended an elderly school with those who did not. For systematic sampling, the sampling interval was determined using the formula I = N/n, where “I” represents the sampling interval, “N” is the total population (11,254), and “n” is the sample size (902). This calculation resulted in a sampling interval of approximately 13 (I = 11,254/902 ≈ 12.5 ≅ 13). Therefore, we included every 13th elderly person in the population who met the eligibility criteria. A random starting point between 1 and 13 was selected using a random number generator, and from this random start, every 13th elderly person in the population who met the eligibility criteria was included in the sample.

**Figure 1.**
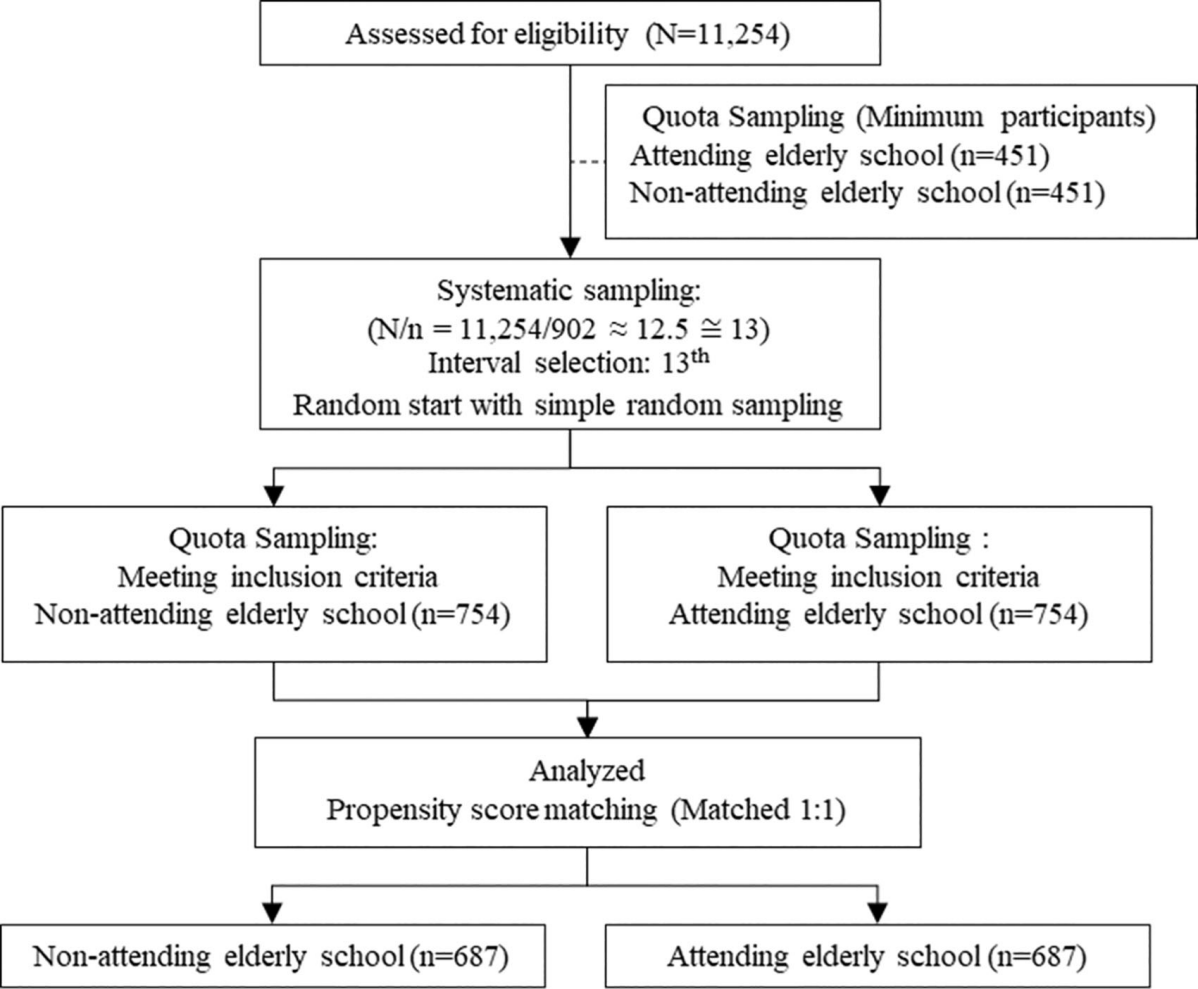
Participant flow diagram (CONSORT).

### Data collection

Data were collected through face-to-face interviews conducted from December 2022 to June 2023. Prior to data collection, the research team, composed of the lead investigator and five experienced healthcare workers in geriatric care, underwent one week comprehensive training. This training covered the study protocol, interview techniques, and participant selection criteria. During data collection, the research team rigorously adhered to inclusion and exclusion criteria for each potential participant.

All participants were provided with written informed consent before the interview process began. We thoroughly explained the study’s objectives, methods, potential risks and benefits, and participants’ rights. Each participant received and signed a written consent form, confirming their voluntary participation. Confidentiality was assured, and participants were informed of their right to withdraw from the study at any time without consequences. To ensure data quality, the lead researcher provided ongoing supervision and support to the research assistants throughout the data collection process.

***Measurement***

The questionnaires were used for data collection as follows: part 1: Individual characteristics, part 2: the Activities of Daily Living (ADLs), and part 3: the World Health Organization Quality of Life – BREF (WHOQOL-BREF).

**Part 1**, included 12 questions assessing demographic and socioeconomic factors, including age, gender, educational level, monthly income of the elderly person, employment, number of family members, living arrangement, illnesses, caregiver, and ADLs. Additionally, participation in an elderly school program was recorded.

**Part 2**, The Barthel Index, an ordinal scale with 10 items, was used to measure ADL performance. Each item, encompassing activities like feeding, toileting, bathing, dressing, and mobility, is scored on a 0-2 scale, reflecting the degree of assistance required. The total score, ranging from 0 to 20, indicates functional capability, with lower scores suggesting greater disability. ^48^ The Cronbach’s alpha was 0.83.

**Part 3**, we used WHOQOL-BREF (Thai version), in which there are 26 questions, ranging from 26 to 130. This questionnaire contains four domains (physical health, psychological health, social relationships, and environment). According to a 5-point Likert scale, each of these domains was scored. ^49^ Following the T-score, raw scores were transformed into a T-score with a range of 20 to 80, with higher scores denoting better QoL in each domain. The WHOQOL-BREF questionnaire showed a Cronbach’s alpha of 0.80.

### Treatment variable

The Elderly School program in Thailand operates on a two-semester basis, with each semester consisting of 50 hours (3 hours per week). This collaborative initiative brings together diverse stakeholders, including the government, Local Administrative Organizations (LAOs), local communities, the private sector, and network partners. Their combined contributions ensure the provision of a wide range of activities and essential facilities for program participants.

The core curriculum for this learning process adheres to the established standards set by the Ministry of Social Development and Human Security. Quality control of teachers and curriculum implementation falls under the responsibility of LAOs, ensuring adherence to national guidelines. However, recognizing the importance of contextually relevant learning, the program allows for flexibility. Additional activities can be specifically tailored to cater to the unique needs and interests of each participating community. This customization plays a crucial role in enhancing engagement and ensuring the program addresses the specific challenges and opportunities faced by local senior citizens. ^29^ In this study, we operationalize the elderly school program as the treatment variable. Individuals who attend more than 80% of classes throughout the program are as “attending elderly school” while those who do not attend classes regularly are “non-attending elderly school”.

The elderly school curriculum consists of physical and mental health, social, economic, and environmental domains. The content can be delivered through workshops, seminars and training. LAOs is the main source for providing staff, financial, and material support, as detailed in Table 1.

**Table 1.** The elderly school curriculum.

Subjects	Content
Physical and mental health	Focusing on understanding and coping with the evolving health needs of elderly populations requires a multifaceted approach that addresses various dimensions of QoL. This encompasses physical, oral, and mental health, nutrition, alongside chronic disease management and responsible medication use. Recognizing the potential value of complementary and integrative practices, such as traditional Thai massage and natural therapies, can further enhance the health care provided to older adults.
Social	This subject tackles the crucial task of adapting social activities to better suit the functional abilities of older adults, covering the topics of laws and benefits, aging and living, knowledge exchange, religious principles, meditation, local wisdom transmission, volunteering, technology, music, singing, rhythm activities, and preserving local traditional cultures. The approach emphasizes tailoring activities to individual needs, considering physical limitations, cognitive abilities, sensory impairments, and social and emotional needs.
Economics	Empowering older adults with the knowledge and skills necessary to achieve financial security and improve their overall QoL, the subject covers such areas as Household Accounting; Sufficiency Economy; Craft Development and Income Generation; Vocational Reskilling and Upskilling, etc.
Environmental	This subject delves into the crucial topic of raising awareness and shaping positive attitudes towards the environment among older adults. It focuses specifically on environment development, encompassing the creation and maintenance of environments, housing, and infrastructure that promote and enhance their QoL. By fostering environmental awareness, promoting safe and accessible environments, and collaborating with key stakeholders, the subject empowers older adults to live healthier, more fulfilling lives while contributing to the creation of sustainable communities for all.

### Covariates

**Figure 2.**
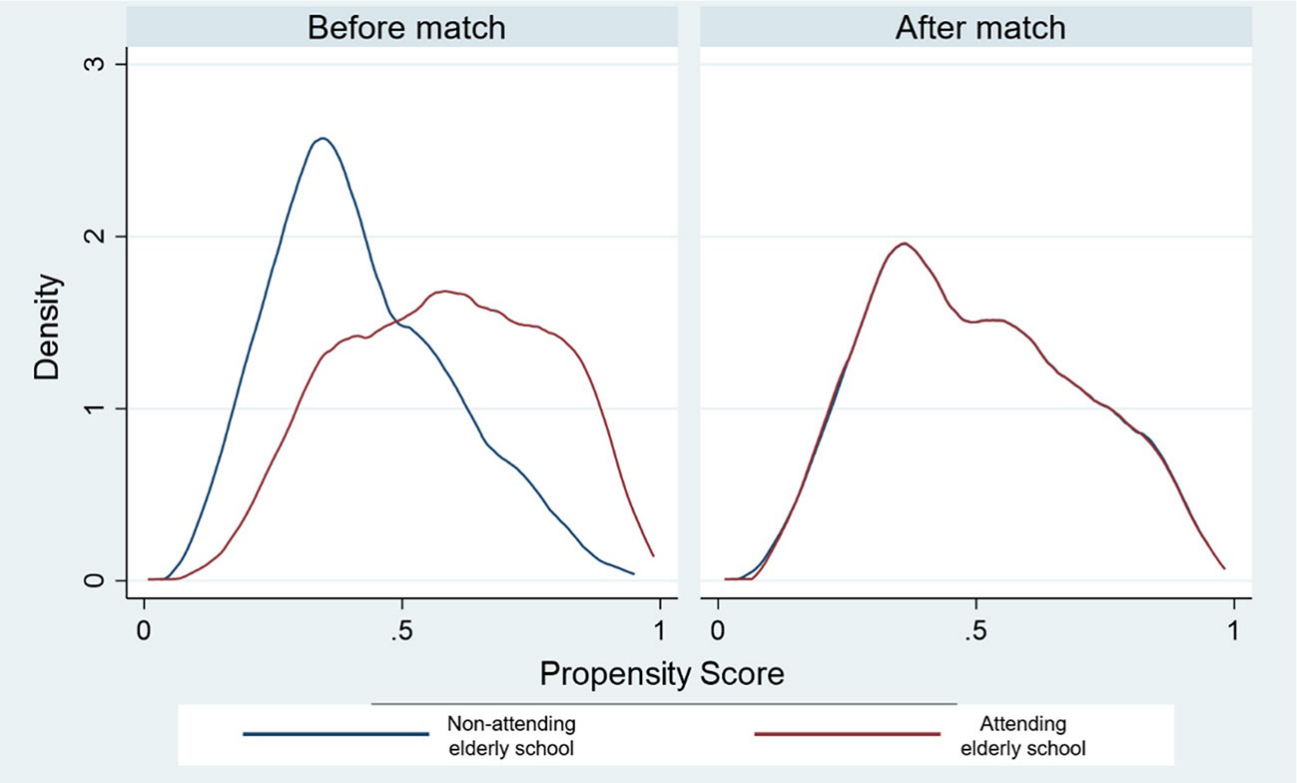
Density of propensity scores before and after match between non-attending and attending the elderly school.

This study employed PSM to address potential selection bias. Demographic variables were considered to calculate the predicted probability of covariates (gender, age, educational level, monthly income, employment, number of family members, living arrangement, illnesses, caregiver, and ADLs) for each individual. A balancing score of the propensity score, and distribution of measured baseline covariates between elderly people who attend elderly school and those who do not attend were compared in Table 2 and Figure 2.

**Table 2.** Demographic characteristics and covariate balancing by standardized difference of non-attending and attending the elderly school.

Factor	Non-attending elderly school n = 687		Attending elderly school n = 687		Standardized difference	
	N	%	N	%	Raw	Matched
Gender						
Male	213	31.00	146	21.25		
Female	474	69.00	541	78.75	0.22	- 0.02
Age (years) (Mean (SD))	67.75 (5.16)		68.66 (4.78)		0.18	0.01
Educational level						
No Education	94	13.68	30	4.37		
Primary school	413	60.12	286	41.63	- 0.38	- 0.06
Secondary school	72	10.48	136	19.80	0.26	0.01
Higher than Vocational Certificate	108	15.72	235	34.21	0.44	0.03
Monthly income (Baht)						
20,000 or less	539	78.46	464	67.54		
Higher than 20,001-30,000	148	21.54	223	32.46	0.25	0.04
Employment						
Unemployed	174	25.33	113	16.45		
Employed	513	74.67	574	83.55	0.22	0.01
Number of family members						
Mean (SD)	3.69 (1.53)		3.75 (1.38)		0.04	- 0.01
Living arrangement						
Living alone	30	4.37	40	5.82		
With family	465	67.69	450	65.50	- 0.05	- 0.04
With a partner	175	25.47	177	25.76	0.01	0.04
With relatives	15	2.18	12	1.75	- 0.03	- 0.04
With friend	2	0.29	8	1.16	0.10	0.01
Hypertension						
No	357	51.97	408	59.39		
Yes	330	48.03	279	40.61	- 0.15	- 0.02
Diabetes						
No	516	75.11	575	83.70		
Yes	171	24.89	112	16.30	- 0.21	- 0.03
Heart disease						
No	676	98.40	682	99.27		
Yes	11	1.60	5	0.73	- 0.08	0.08
Caregiver						
No	140	20.38	153	22.27		
Yes	547	79.62	534	77.73	- 0.46	- 0.09
The activities of daily living (ADLs) Mean (SD)	18.46 (1.98)		18.90 (1.60)		0.16	- 0.01

### Statistical analysis

Categorical variables were described by frequency and percentage (%). Continuous variables were described by mean and standard deviation (SD). PSM was applied to match between the elderly people who attended the elderly school and those who did not. A regression model based on characteristic variables was used to compute propensity scores for each participant. K-nearest neighbor 1:1 matching model with no replacement and no callipers was employed as the propensity score matching estimator in Python. The standardized difference was used to examine the balance of covariate distribution between groups, which was independent of the unit of measurement. It allows comparison between variables with different units of measurement to calculate the propensity scores with a maximum standardized difference of 10%. Then, the average treatment effects of the population (ATE) were analyzed to investigate the effect size of the elderly schools on QoL, and multiple linear regression was analyzed to investigate the association between elderly school attendance and QoL using Stata 17.0. The level of two-sided statistical significance was set at p < 0.05.

## Results

There were 1,508 participants who completed the interview in this study. Following PSM, the sample size remained at 1,374 individuals, with 687 in each group (elderly school non-attending group and attending group).

### Comparison of the demographic characteristics of elderly people between non-attending and attending the elderly school

There were 687 participants in each group, with 474 (69.00%) elderly women in the non-attending elderly school group and 541 (78.75%) in the attending group. The average age of elderly people in the non-attending group was 67.75 (SD = 5.16) and in the school-attending group was 68.66 (SD = 4.78). Most participants had completed primary school, monthly income of less than 20,000 baht, and were employed in both groups, as over half of the participants lived with their families and had caregivers to take care of them. The results indicate the standardized difference was less than 10% on all covariates, which identified improved balance between the two groups ( Table 2).

### The effect of elderly school on quality of life

The average overall QoL of elderly people in the attending elderly school group was 57.50 (SD = 7.53) and that of those in the non-attending elderly school group was 44.40 (SD = 7.11). The ATE of overall QoL was 10.67 (95% CI: 9.67–11.67, p-value <0.001), indicating a positive effect of attending the elderly school on QoL. Additionally, the ATE between the two groups for QoL domains: physical health, psychological, social relationships, and environmental dimensions were 8.89, 8.17, 6.37, and 8.41, respectively. There was a statistically significant difference in each QoL domain between the two groups ( Table 3).

**Table 3.** The average treatment effects (ATE) on quality of life after PSM.

Outcome	Mean (SD)		ATE (scores)	SE	95% CI of ATE	p-value
	Non-attending elderly school n = 687	Attending elderly school n = 687				
Overall QoL	44.40 (7.11)	57.50 (7.53)	10.67	0.51	9.67 – 11.67	<0.001
Physical health	45.40 (8.09)	56.22 (8.51)	8.89	0.60	7.71 – 10.08	<0.001
Psychological	45.61 (8.41)	57.32 (8.57)	8.17	0.56	7.06 – 9.27	<0.001
Social relationships	46.64 (8.80)	54.46 (9.51)	6.37	0.61	5.16 – 7.58	<0.001

### The association between elderly school attendance and quality of life

A multiple linear regression was conducted to assess the relationship between factors and QoL. As presented in Table 4, QoL was significantly associated with gender, educational level, monthly income, employment, caregiver, and elderly school attendance (F (8, 1365) = 251.88, p < 0.001 $R^{2}\;$ = 0.5962 and adjusted $R^{2}$ = 0.5938). Notably, elderly school attendance showed a strong association with QoL. The unstandardized beta coefficient for elderly school attendance was 10.811. Higher education levels among elderly people were also associated with higher QoL. The unstandardized beta coefficients for primary, secondary, and education higher than vocational certificates are 5.472, 10.090, and 10.157, respectively. This suggests that, for each elderly person with a higher educational level, the QoL score increases by the respective unstandardized beta coefficient ( Table 4).

**Table 4.** The association between elderly school attendance and quality of life.

Factors (n=1,374)	Full model			Final model		
	Unstandardized		Standardized	Unstandardized		Standardized
	*B*	*SE of B*	β	*B*	*SE of B*	β
Constant	29.918	3.118				
Age (years)	0.052	0.034	0.027			
Elderly school attendance						
No	Ref.					
Yes	10.674	0.098	0.544 *	10.811	0.360	0.550 *
Gender						
Male	Ref.					
Female	1.812	0.392	0.081 *	1.754	0.389	0.078 *
Educational level						
No Education	Ref.					
Primary school	5.478	0.644	0.279 *	5.472	0.619	0.279 *
Secondary school	9.965	0.822	0.364 *	10.090	0.803	0.368 *
Higher than vocational certificate	10.04	0.840	0.443 *	10.157	0.824	0.448 *
Monthly income (Baht)						
20,000 or less	Ref.					
Higher than 20,001-30,000	1.380	0.580	0.062 **	1.313	0.576	0.059 *
Employment						
Unemployed	Ref.					
Employed	2.284	0.438	0.095 *	2.270	0.434	0.094 *
Number of family members	0.168	0.119	0.025			
Living arrangement						
Living alone	Ref.					
With family	- 0.927	0.794	- 0.045			
With a partner	- 1.582	0.831	- 0.070			
With relatives	- 0.103	1.427	- 0.001			
With friend	- 0.928	2.124	- 0.008			
Hypertension						
No	Ref.					
Yes	- 0.306	0.365	- 0.015			
Diabetes						
No	Ref.					
Yes	- 0.499	0.454	- 0.021			
Heart disease						
No	Ref.					
Yes	- 0.629	1.591	- 0.007			
Caregiver						
No	Ref.					
Yes	1.528	0.418	0.064 *	1.574	0.414	0.066 *
Elderly school attendance						
No	Ref.					
Yes	10.674	0.098	0.544 *	10.811	0.360	0.550 *
The activities of daily living (ADLs)	0.066	3.118	0.012			

* p < 0.001.

** p < 0.005.

## Discussion

This study investigates the effect of elderly school policy on QoL across its four domains: physical health, psychological, social relations, and environment. Furthermore, the associations between the factors and QoL were identified. Notably, in our study, the QoL of elderly people in the attending elderly school group was higher than that of those in the non-attending elderly school group, and the ATE indicated a positive effect of attending the elderly school on QoL. This finding is comparable with previous research on elderly schools in the northern and eastern regions of Thailand, which similarly found higher QoL among attending elderly school groups compared to non-attending elderly school groups. ^50^^–^^52^ Physical health was shown to be the highest domain in ATE, followed by the psychological, environmental, and social relationships domains, all of which had similar values except for social relationships. This result reflects the fact that the elderly people emphasized their health deterioration with increasing age and the burden of diseases. ^53^^,^^54^

Moreover, our results resonate with broader international research highlighting the positive impact of interventions based on the concept of lifelong learning. Our finding was consistent with a study in Portugal that investigated the effect of participation in community intervention programs and indicated that QoL was better in the physical domain than others. ^55^ Similarly, a systematic literature review on the effects of later-life formal education on the QoL of elderly people also revealed a positive impact on QoL. ^56^ Additionally, a study in Canada regarding community-based participation in programs for mental health found a positive impact on psychological well-being, particularly in the context of non-formal lifelong learning ^57^ and the elderly individuals attending evening schools for lifelong learning in Korea experienced positive well-being impacts, including in the environmental dimension. ^58^

Our study further strengthens the evidence for a significant positive association between elderly school attendance and QoL after adjusting for other independent variables in a multiple linear regression model. This finding mirrors that of a study in Rayong Province, Thailand, which similarly identified the participation of senior citizens in educational management in elderly schools as a positive predictor of QoL, demonstrating the program’s potential to elevate QoL among attendees. ^59^ Furthermore, our results confirmed that sociodemographic factors, including gender, educational level, monthly income, and employment, were significant factors in QoL. For instance, this result was consistent with a study conducted in a rural area of the northern and northeast region of Thailand that found sociodemographic factors related to the elderly’QoL. ^60^^,^^61^ Additionally, male gender, lack of education, and lower economic status are associated with low QOL of elderly people in rural area, India. ^62^ In Korea, higher monthly income of elderly people had a positive effect on QoL. ^63^ Furthermore, the presence of a caregiver was a positively significant factor in the QoL of elderly people. This result was consistent with a study conducted by developing caregivers’ potential to improve the QoL for the elderly in the southern region of Thailand. ^64^ It is important to note that the specific factors associated with QoL vary across these studies. This variability can be attributed to differences in research methodology.

### Strengths and limitations

This study displays several key strengths that contribute to its significance and pave the way for future research. Firstly, the utilization of PSM and estimation of average treatment effects (ATEs) strengthens the study’s internal validity. By creating a setting that mimics real-world conditions, PSM facilitates a comparison between elderly school attendees and non-attendees, minimizing selection bias. Furthermore, it stands as a pioneering analysis of the elderly school policy’s influence on QoL. This comprehensive approach provides a nuanced understanding of the policy’s multifaceted impact, surpassing previous research that primarily focused on program development.

However, this study acknowledges several limitations. Firstly, geographical scope: conducted in a single district, the results may not directly generalize to other populations or cultural contexts. Nevertheless, Lomkao district with its significant elderly population serves as a valuable study, offering important insights applicable to similar demographics. Secondly, cross-sectional design: While providing valuable data on QoL and its association with elderly school attendance, the cross-sectional design restricts the ability to establish causal relationships. Longitudinal studies are needed to definitively examine the impact of the policy over time. Future studies may benefit from incorporating additional assessments and corroborative data sources.

### Policy implications and recommendations

The findings of this study offer profound policy implications and recommendations towards optimizing Thailand’s approach to senior citizens through lifelong learning initiatives like the elderly school program. 1) Integration into broader health promotion strategies: recognizing the significant QoL improvements associated with elderly school attendance underscores the imperative to embed this program within broader health promotion strategies for aging populations. This integration can leverage synergies between lifelong learning and other QoL initiatives. 2) Fostering continuous education policies: our results further advocate for the development of dedicated policies specifically aimed at supporting and promoting continuous education among senior citizens. Such policies could range from targeted funding mechanisms for elderly school expansion to incentivizing the creation of age-friendly learning materials and curricula. 3) Collaborative action for strong support systems: to maximize the impact of elderly schools and similar initiatives, we propose enhanced collaboration and awareness promotion among relevant authorities and stakeholders. This could involve partnerships between government agencies, community organizations, and the private sector to foster robust support systems for aging societies, prioritizing elderly schools, health promotion programs, and QoL initiatives. 4) Rural-specific models for inclusive QoL enhancement: recognizing the unique needs and challenges of rural elderly populations, we strongly recommend the development and promotion of elderly school models specifically tailored for rural communities. These models may require adaptations to curriculum content, delivery methods, and resource allocation to ensure equitable access and maximize QoL improvements for all senior citizens regardless of location. By embracing these policy implications and recommendations, Thailand can leverage the power of lifelong learning to establish a comprehensive and inclusive framework for healthy aging within its growing senior population.

More importantly, to broaden the context and enhance the study’s global relevance, it is essential to consider how the findings from Thailand’s elderly school policy could inform similar initiatives in other countries with aging populations. Many middle-income countries facing rapid demographic aging share similar socioeconomic and healthcare challenges, including limited access to educational and social support services for older adults. Developing elderly school models tailored to rural communities, as proposed in this study, could serve as a valuable framework for other nations seeking to improve the quality of life among their senior citizens. For instance, countries in Southeast Asia, Latin America, and Eastern Europe, where rural aging populations are growing, could adopt and adapt Thailand’s approach by modifying curriculum content, delivery methods, and resource allocation to reflect local cultural, economic, and infrastructural conditions. This cross-cultural application would not only promote healthy aging but also foster social inclusion and lifelong learning on a global scale.

## Conclusion

The present study underscores the elderly school policy as a potentially impactful health promotion intervention, offering valuable insights into its effectiveness. Notably, the study reveals significantly higher overall QoL, and improvements across specific domains, among senior citizens attending elderly schools compared to their non-attending counterparts. Additionally, attendance at elderly schools emerges as a significant predictor of QoL, highlighting the program’s direct contribution to QoL. While certain sociodemographic factors also exhibit significant associations with QoL, the study’s findings provide compelling evidence that the lifelong learning policy embodied by elderly schools has demonstrably enhanced the QoL of senior citizens in Thailand.

To maximize the program’s potential, policymakers should prioritize robust support efforts across various domains. Crucial areas include adequate human resources, dedicated financial resources, well-equipped facilities, and effective management structures. Furthermore, encouraging collaboration with LAOs, communities, and the private sector can play a vital role in promoting and expanding the implementation of elderly schools. By prioritizing such strategic investments and collaborative efforts, Thailand can leverage the powerful potential of lifelong learning initiatives to significantly improve the QoL of its aging population.

### Ethical and consent

This study received ethical approval from the Institutional Review Board (IRB) of Phetchabun Hospital (approval number IEC-20-2565, date: November 9, 2022). The study adhered to the ethical principles outlined in the Declaration of Helsinki, The Belmont Report, CIOMS Guideline, and International Conference on Harmonization in Good Clinical Practice (ICH-GCP). Written informed consent was obtained from all participants before the interview process began. Participants were informed that they could withdraw from the study at any time without providing a reason. The confidentiality of participant data was ensured throughout the entire study.

## Data Availability

Figshare: Effect of elderly school policy on quality of life among Thailand’s senior citizens, https://doi.org/10.6084/m9.figshare.25894831.v2. ^65^ The project contains the following underlying data:
• Data.xlsx • Ethical considerationsap • Participant information sheets and consent forms • Questionnaire Data are available under the terms of the Creative Commons Attribution 4.0 International license (CC-BY 4.0). Questionnaire, ethical considerations, participant information sheets and consent forms were provided in data availability https://doi.org/10.6084/m9.figshare.25894831.v2. ^65^
