# Supplementary material for: Effect of Elderly School Policy on Quality of Life among Thailand’s Senior Citizens: A Propensity Score Matching Approach
Source: F1000Res. 2025 Mar 31;13:735. Originally published 2024 Jul 3. [Version 2] doi: 10.12688/f1000research.151221.2 (PMC11831888; doi:10.12688/f1000research.151221.2)
Supplement: Peer review history

# Reviewer report: Reviewer response for version 2

**DOI:** 10.5256/f1000research.179137.r374503

**Published:** 2025-04-16

**Authors:** Aye Sandar Mon

**Affiliations:** 1 University of Public Health, Yangon, Myanmar

No further comments from my side. All my previous comments have been addressed in the revised version.

Is the work clearly and accurately presented and does it cite the current literature?

Yes

If applicable, is the statistical analysis and its interpretation appropriate?

Partly

Are all the source data underlying the results available to ensure full reproducibility?

Yes

Is the study design appropriate and is the work technically sound?

Yes

Are the conclusions drawn adequately supported by the results?

Yes

Are sufficient details of methods and analysis provided to allow replication by others?

Yes

Reviewer Expertise:

My areas of research include maternal health and other public health-related topics, epidemiological studies in communicable and non-communicable diseases, and the application of advanced statistical models in various types of research.

I confirm that I have read this submission and believe that I have an appropriate level of expertise to confirm that it is of an acceptable scientific standard.

# Reviewer report: Reviewer response for version 1

**DOI:** 10.5256/f1000research.165851.r310490

**Published:** 2025-02-13

**Authors:** Ueamporn Summart

**Affiliations:** 1 Faculty of Nursing, Roi Et Rajabhat University, Tha Muang, Thailand

Title: Effect of Elderly School Policy on Quality of Life among Thailand’s Senior Citizens: A Propensity Score Matching Approach.

 After conducting a thorough analysis of all your articles, this manuscript seeks to assess the impact of geriatric education on the quality of life of older individuals. The findings of this article are essential for informing government policies aimed at enhancing the quality of life for this specific demographic. The author employs statistical tools to mitigate biases and apply the results to favorable health outcomes. However, there are certain aspects that leaders should comprehend and acknowledge:

 1. Calculation of the required sample size: Please provide further elaboration on the concept of "effect size = 0.02," including information on how to compute it and the specific value obtained from the pilot study.

 2. Study samples: This study employed quota and systematic simple random sampling techniques, which can be considered a two-step sampling process or referred to as multi-stage random sampling. In this section, I suggest that the author include a CONSORT diagram detailing the process of participant enrollment in the trial, which would enhance the reader's comprehension.

 3. Results: This study employed the beta coefficient to represent the extent of the study's influence. Given that this study used multiple linear regression to assess the impact of covariates on quality of life (QOL), it is necessary to report the mean difference.

Is the work clearly and accurately presented and does it cite the current literature?

Yes

If applicable, is the statistical analysis and its interpretation appropriate?

Yes

Are all the source data underlying the results available to ensure full reproducibility?

Yes

Is the study design appropriate and is the work technically sound?

Yes

Are the conclusions drawn adequately supported by the results?

Yes

Are sufficient details of methods and analysis provided to allow replication by others?

Yes

Reviewer Expertise:

Nursing and Public Health

I confirm that I have read this submission and believe that I have an appropriate level of expertise to confirm that it is of an acceptable scientific standard.

# Response

**Published:** 2025-03-23

**Authors:** Worapath Kratoo

**Affiliations:** College of Public Health Scien, Chulalongkorn University, Bangkok, Bangkok, Thailand

We sincerely appreciate the reviewer's insightful comments, which have significantly helped us to improve the clarity of our manuscript. We have addressed each point as follows:

**Comment 1: Calculation of the Required Sample Size: Effect Size**

- **Response: **An effect size of 0.02 in the sample size calculation, derived from a pilot study comparing QoL between the means of the two groups (attending and not attending elderly school) divided by the standard deviation.

**Comment 2: Study Samples: Sampling Process and CONSORT Diagram**

- **Response:** We have already added CONSORT diagram

**Comment 3: Results: Beta Coefficients and Mean Differences**

- **Response:** In our analysis, we have chosen to present Average Treatment Effects (ATE) as our primary measure of impact, as these values provide a more robust estimate of causal effect by accounting for potential confounding variables. The ATEs reported in our results section represent the estimated average effect of each variable on quality of life after controlling for other covariates in the model. We believe this approach is more appropriate for our study design than unadjusted mean differences because it addresses potential selection bias and confounding factors that could influence the outcomes.

We believe that these revisions have addressed the reviewer's concerns and significantly improved the manuscript. We are grateful for the opportunity to enhance the quality of our work.

# Reviewer report: Reviewer response for version 1

**DOI:** 10.5256/f1000research.165851.r302071

**Published:** 2024-09-20

**Authors:** Andrew Kweku Conduah

**Affiliations:** 1 University of Professional Studies, Madina, Ghana

**Effect of Elderly School Policy on Quality of Life among Thailand’s Senior Citizens: A Propensity Score Matching Approach.**

**Summary of the Manuscript:**

 This manuscript evaluates the impact of Thailand’s 'elderly school' initiative on the quality of life (QoL) among senior citizens using a propensity score matching (PSM) approach. The study, conducted in Phetchabun province, found that attending the elderly school significantly improved QoL compared to non-attendees, offering a beacon of hope for the elderly population. The research also identified several factors positively associated with QoL, including gender, income, employment, caregiving, and education.

**Evaluation of the Introduction**

**Global Context:** The introduction effectively situates the research within the global context by highlighting the significant increase in the elderly population worldwide. It references the international trend of an ageing population, emphasizing the impact on healthcare systems. Statistics about the projected growth of the elderly population from 2015 to 2050 and the concentration in middle-income countries provide a strong foundation for understanding the global scale of the issue. The comparison with other regions, such as South India and Myanmar, also enhances the global perspective.

**Research Problem Articulation and Justification:** The research problem is well-articulated and justified. The introduction clearly identifies the urgent need for effective interventions to improve senior citizens' quality of life (QoL) in the face of the ageing population in Thailand. The discussion on the disparities in QoL between urban and rural areas, as well as the specific context of Lomkao district, further justifies the need for the study. The problem is contextualized by noting the lack of comprehensive evaluations of the elderly school initiative’s impact, which underscores the significance of the research.

**Objectives:** The introduction does not explicitly state the research objectives. While the problem is well-defined and justified, and the gap in the literature is highlighted, the study's specific aims should be more clearly articulated. For instance, the introduction could explicitly state that the study aims to evaluate the causal effect of the elderly school initiative on QoL using propensity score matching (PSM) and to investigate the association between various factors and QoL among senior citizens.

**Opinion:** The introduction provides a robust and comprehensive background for the study. It effectively places the research in a global context, identifies the problem, and justifies the need for the study. However, the specific research objectives should be clearly stated to strengthen the introduction. This will provide readers with a clear understanding of the study’s aims and how it intends to address the identified gap in the literature. Additionally, expanding the discussion on similar interventions in other regions could further enhance the global context and relevance of the research. Overall, the introduction sets a solid foundation for the study but would benefit from a more explicit articulation of the research objectives.

**Methodology**

 1. **Research Design**

**Cross-Sectional Study:**

- **Appropriateness:** The cross-sectional design is suitable for simultaneously assessing the prevalence of characteristics or conditions. This study is used to compare the quality of life (QoL) of elderly individuals who attended and did not attend an elderly school. This design effectively examines differences in QoL and determines associations with the elderly school program.

2. **Sample Size**

**Sample Size Calculation:**

- **Appropriateness:** The sample size calculation is based on continuous data and considers effect size, power, predictors, and significance level. The study's sample size of 901 participants is derived from these calculations, ensuring sufficient statistical power for detecting meaningful differences.

3. **Sampling Technique**

**Quota and Systematic Sampling:**

- **Appropriateness:** Combining quota sampling with systematic sampling ensures a representative sample of elderly individuals attending and not attending the elderly school. Quota sampling ensures demographic representation, while systematic sampling introduces randomness into the selection process.

4. **Data Collection**

**Face-to-Face Interviews:**

- **Appropriateness:** Face-to-face interviews effectively collect detailed and accurate data concerning literacy or language barriers. The research team’s training ensures consistency and reliability in data collection.

5. **Measurement**

**Instruments Used:**

- **Demographic and Socioeconomic Factors:** Detailed questionnaires assess various factors relevant to QoL.
- **Activities of Daily Living (ADLs):** The Barthel Index is an established tool for measuring ADL performance, suitable for evaluating functional capacity.
- **WHOQOL-BREF:** This validated instrument assesses QoL across multiple domains. Using the Thai version ensures cultural and linguistic relevance.

6. **Treatment Variable**

**Elderly School Program:**

- **Appropriateness:** The definition and criteria for attendance in the elderly school program are clear and relevant to the study's objectives. Participants are categorised based on their attendance, which aligns with the study’s aim to evaluate the program’s impact.

7. **Covariates and Propensity Score Matching (PSM)**

**PSM for Addressing Selection Bias:**

- **Appropriateness:** PSM is a robust method for controlling confounding variables and ensuring balanced groups. Demographic and socioeconomic factors are used as covariates, enhancing comparability between attendees and non-attendees of the elderly school.

8. **Statistical Analysis**

**Analysis Methods:**

- **Appropriateness:** The use of PSM, standardised differences, and multiple linear regression is appropriate for analysing the effect of the elderly school program on QoL. These methods effectively control for confounding and assess the treatment's impact.

**Results**

***Demographic Characteristics of the Participants***

 A total of 1,508 participants completed the interview. After Propensity Score Matching (PSM), 1,374 individuals remained, with 687 participants in the non-attending and attending elderly school groups. In the non-attending group, 474 (69.00%) were women, and 541 (78.75%) were women in the attending group. The average age in the non-attending group was 67.75 years (SD = 5.16), while in the attending group, it was 68.66 years (SD = 4.78). Most participants in both groups had completed primary school, had a monthly income of less than 20,000 baht, and were employed. More than half of the participants lived with their families and had caregivers. The standardised difference was less than 10% for all covariates, indicating improved balance between the two groups (Table 2).

***Quality of Life Outcomes***

 The average overall Quality of Life (QoL) score was 57.50 (SD = 7.53) in the attending group and 44.40 (SD = 7.11) in the non-attending group. The Average Treatment Effect (ATE) of attending elderly school on overall QoL was 10.67 (95% CI: 9.67–11.67, p < 0.001). The ATEs for QoL domains were as follows: physical health (8.89), psychological (8.17), social relationships (6.37), and environmental (8.41). Each domain showed a statistically significant difference between the two groups (Table 3).

***Associations Between Elderly School Attendance and QoL***

 Multiple linear regression analysis revealed that QoL was significantly associated with gender, educational level, monthly income, employment, caregiver presence, and elderly school attendance (F (8, 1365) = 251.88, p < 0.001, R² = 0.5962). Elderly school attendance had a strong positive association with QoL, with an unstandardised beta coefficient of 10.811. Higher education levels were also associated with higher QoL scores (Table 4).

**Discussion**

 This study examined the impact of attending an elderly school on QoL across four domains: physical health, psychological well-being, social relationships, and environment. The findings revealed that elderly school attendance significantly improved QoL compared to non-attendance. The positive effect of elderly school attendance was consistent with previous research in different regions of Thailand, which also found higher QoL among school attendees. The highest impact was observed in the physical health domain, followed by the psychological, environmental, and social relationships domains.

 The study's results align with international research, highlighting the benefits of lifelong learning for elderly populations. Studies from Portugal, Canada, and Korea have observed similar positive impacts on QoL, underscoring the global relevance of these findings.

**Strengths and Limitations**

 The study's strengths include using PSM to enhance internal validity and provide a realistic comparison between groups. It is one of the first to analyze the elderly school policy's impact on QoL comprehensively.

 However, limitations include the study's geographical scope, as it was conducted in a single district, limiting generalizability. The cross-sectional design also restricts the ability to establish causality, and future longitudinal studies are needed to confirm these findings.

**Policy Implications and Recommendations**

 The findings suggest important policy implications for optimizing Thailand's approach to ageing populations. The study recommends integrating elderly schools into broader health promotion strategies, developing continuous education policies for seniors, fostering stakeholder collaboration, and creating rural-specific models to enhance QoL for elderly populations in similar contexts.

 The conclusion is well-structured and effectively summarizes the essential findings and implications of the study. Here’s a brief evaluation:

1. **Clear Summary of Findings**: The conclusion restates the primary outcomes, particularly the positive impact of elderly school attendance on Quality of Life (QoL). It effectively highlights the significance of these schools in enhancing overall QoL among senior citizens in Thailand.
2. **Implications for Policy**: The discussion on policy implications is well-founded. By recommending robust support across various domains (human resources, finances, facilities, and management), the conclusion addresses practical steps that policymakers can take to enhance the program's effectiveness.
3. **Encouragement of Collaboration**: A strategic recommendation is to emphasise collaboration with local administrative organisations (LAOs), communities, and the private sector. This suggests a comprehensive approach to expanding the program and ensures the reader understands the context needed for success.
4. **Forward-looking Perspective**: The conclusion reflects on the current findings and looks ahead, suggesting how Thailand can further leverage these initiatives to improve the QoL of its ageing population. This gives the study a forward-looking angle, which is valuable in research conclusions.

**Minor Suggestions for Enhancement**:

- **Reinforce Study Limitations**: Depending on what was discussed earlier in the paper, briefly mentioning any limitations or areas for future research here might be helpful. This adds balance and shows a critical engagement with the study’s scope.
- **Broaden the Context**: While the focus on Thailand is appropriate, you could briefly mention how these findings might inform similar initiatives in other countries, especially those with ageing populations, to widen the study's impact.

Overall, the conclusion is appropriate, but adding a sentence or two to address limitations or broader implications could make it even stronger.

Is the work clearly and accurately presented and does it cite the current literature?

Yes

If applicable, is the statistical analysis and its interpretation appropriate?

Yes

Are all the source data underlying the results available to ensure full reproducibility?

Yes

Is the study design appropriate and is the work technically sound?

Yes

Are the conclusions drawn adequately supported by the results?

Yes

Are sufficient details of methods and analysis provided to allow replication by others?

Yes

Reviewer Expertise:

§  Population Estimates and Projections.§  Population Health and Mortality- Health Emergencies & Preparedness, Ageing & Intergeneration Relations, and Non-Communicable Disease & Health Projection (Physical Activity, Obesity, Tobacco Control, etc.)§  Business Demography§  Business Policy and Strategy§  Business Management§  Public Administration, Entrepreneurial Innovation

I confirm that I have read this submission and believe that I have an appropriate level of expertise to confirm that it is of an acceptable scientific standard.

# Response

**Published:** 2025-03-23

**Authors:** Worapath Kratoo

**Affiliations:** College of Public Health Scien, Chulalongkorn University, Bangkok, Bangkok, Thailand

We sincerely appreciate the reviewer's insightful comments, which have significantly helped us to improve the clarity of our manuscript. We have addressed each point as follows:

**Suggestion 1: Reinforce Study Limitations**

- **Response: **Thank you for your thoughtful suggestion to reinforce the study limitations. We would like to note that the manuscript already includes a detailed discussion of the study limitations in the 'Strengths and limitations' section. Specifically, we have addressed geographical limitations (the single-district scope and generalizability concerns), methodological limitations (cross-sectional design and its constraints on establishing causality), and have suggested directions for future research including longitudinal studies and additional assessment approaches. 

**Suggestion 2: Broaden the Contex**t

- **Response: **Thank you for your suggestion to broaden the context. We agree this would strengthen the paper's impact. While our policy implications section currently focuses on the Thai context, we will add a paragraph explicitly discussing how our findings could inform similar initiatives in other countries with aging populations. This addition will highlight the potential transferability of our findings regarding elderly education models to various cultural and socioeconomic contexts globally, particularly in other middle-income countries experiencing rapid demographic aging.

We believe that these revisions have addressed the reviewer's concerns and significantly improved the manuscript. We are grateful for the opportunity to enhance the quality of our work.

# Reviewer report: Reviewer response for version 1

**DOI:** 10.5256/f1000research.165851.r310486

**Published:** 2024-08-23

**Authors:** Aye Sandar Mon

**Affiliations:** 1 University of Public Health, Yangon, Myanmar

**General comment**

- The manuscript is well written and the study findings could contribute to the substantial policy implication.

**Specific comments**

- In sampling technique, the researchers applied quota and systematic sampling. For systematic sampling, how to calculate the sampling interval was described but I did not find about the random start. Could you please clarify it?
- In data collection method, was any pre-testing conducted? If so, please mention the procedure and the results including the reliability of the questionnaire and participants’ understanding of the question items.
- According to previous literature and evidence from international studies, in addition to the factors you considered as covariates in the statistical model, the social support and mental health status such as depression have been recognized as the important predictors for QoL. Although your questionnaire included the items assessing the social support and mental health status (DASS-21), why weren’t these variables considered as covariates in the statistical model?
- While presenting the results of multiple linear regression in “Table 4”, as the variable “elderly school attendance” is the main factor of interest, this variable should be described first instead of being the second-to-last description.
- I did not find the statistical procedures for assumption checks for multiple linear regression (regression diagnostics) in the 'Methods' or 'Results' sections. Were these tests performed? If not, please check the necessary assumptions, and if they were, please mention the results in the manuscript.

Is the work clearly and accurately presented and does it cite the current literature?

Yes

If applicable, is the statistical analysis and its interpretation appropriate?

Partly

Are all the source data underlying the results available to ensure full reproducibility?

Yes

Is the study design appropriate and is the work technically sound?

Yes

Are the conclusions drawn adequately supported by the results?

Yes

Are sufficient details of methods and analysis provided to allow replication by others?

Yes

Reviewer Expertise:

My areas of research include maternal health and other public health-related topics, epidemiological studies in communicable and non-communicable diseases, and the application of advanced statistical models in various types of research.

I confirm that I have read this submission and believe that I have an appropriate level of expertise to confirm that it is of an acceptable scientific standard, however I have significant reservations, as outlined above.

# Response

**Published:** 2025-03-23

**Authors:** Worapath Kratoo

**Affiliations:** College of Public Health Scien, Chulalongkorn University, Bangkok, Bangkok, Thailand

We sincerely appreciate the reviewer's insightful comments, which have significantly helped us to improve the clarity of our manuscript. We have addressed each point as follows:

**Comment 1: Sampling Technique (Systematic Sampling - Random Start)**

- **Response:** Thank you for pointing out the omission regarding the random start in our systematic sampling procedure. To clarify, we have now added this important information to the sampling technique section. Specifically, we used a random number generator to select a starting point between 1 and 13, and from this random start, we then selected every 13th elderly person in the population who met our eligibility criteria. This random start was essential to ensure the systematic sample maintained randomness properties.

**Comment 2: Data Collection Method (Pre-testing and Reliability)**

- **Response:** We conducted a pilot study for pre-testing, and the reliability of the questionnaire was evaluated using Cronbach's alpha. All items demonstrated reliability coefficients between 0.80-0.83, indicating good internal consistency. These reliability measures have been incorporated into the methodology section.

**Comment 3: Covariates (Social Support and Mental Health)**

- **Response:** The propensity score matching (PSM) analysis, we focused on demographic characteristic variables that enable the matching of similar characteristics, thus maintaining’s model simplicity, focusing on direct effects rather than confounding relationships that could complicate analysis.

**Comment 4: Presentation of Multiple Linear Regression Results (Elderly School Attendance)**

- **Response:** We agree that the variable 'elderly school attendance, being the main factor of interest, should be highlighted. In the revised manuscript, we have reordered the results in Table 4.

**Comment 5: Regression Diagnostics (Assumption Checks)**

- **Response:** There is no multicollinearity, as indicated by the VIF scores, which are all less than 10 in Table 4.

We believe that these revisions have addressed the reviewer's concerns and significantly improved the manuscript. We are grateful for the opportunity to enhance the quality of our work.
